# Application of bilateral crossing C2 laminar screws and screw placement assistant tools for upper cervical spine fixation: technology note

**DOI:** 10.3389/fsurg.2025.1648329

**Published:** 2025-11-04

**Authors:** Hongfei Qi, Haoxuan Feng, Zhong Li, Ming Li, Bo Wu, Chengcheng Zhang, Shichang Liu

**Affiliations:** Honghui Hospital, Xi'an Jiaotong University, Xi'an, China

**Keywords:** C-2 translaminar screws, tool, atlantoaxial stabilization, technique, fixation

## Abstract

**Background:**

Axial lamina screw placement is a new posterior cervical fixation technique that differs from the previously described transarticular screw placement and pedicle screw placement in terms of the risk of causing damage to the vertebral artery. However, the previously reported laminar screw placement technique carries the risk of causing damaging to the screws in the inner wall of the vertebral plate and screw protrusion into the spinal canal. To address this issue, our center has designed a screw placement assistant tool to help surgeons safely insert screws.

**Methods:**

The medical records of 6 patients with upper cervical spine injuries who were admitted to Xi'an Honghui Hospital between March 2021 and February 2022 were retrospectively analyzed to assess the patients' postoperative recovery. Self-designed screw placement assistants were used to insert double cortical laminar screws for posterior cervical fixation. Computed tomography (CT) scanning was performed to observe screw placement after surgery. The clinical and postoperative recovery data of the patients were reviewed, and the clinical efficacy of aids for bicortical screw placement in the axial (C2) vertebral lamina were evaluated.

**Results:**

All patients were followed up for 9–16 months, with an average of 11.5 months. The fractures were satisfactorily fixed and healed. No patients experienced wound infection, internal fixation failure, or secondary surgery. Postoperative CT scans showed that a total of 12 C2 bicortical lamina screws were implanted in 6 patients. The axial lamina screws were well positioned and located in the center of the axial lamina, without any deviation or detachment. There were also no cases in which screws penetrated the medial cortical bone of the lamina or protruded into the spinal canal. No patients experienced vertebral artery injury.

**Conclusions:**

C2 laminar screw placement, as a technique for posterior fixation of the high cervical spine, can be a good choice when transarticular screw and pedicle screw placement are difficult. Our designed screw placement assistance tool can help surgeons safely and accurately insert bicortical cross screws into the axial vertebral lamina, avoiding screw damage to the inner wall of the lamina and spinal canal.

## Introduction

Upper cervical dislocation is usually caused by congenital malformations, trauma, tumors, inflammation, rheumatoid arthritis, and other factors that lead to the loss of normal alignment between the atlas and axis. Upper cervical spine dislocation can cause severe cervical spinal cord injury or respiratory failure, and the complex anatomical structure of the upper cervical spine can be deeply localized, and surgery can be technically challenging and high-risk. With the development of imaging and the diagnostic recognition in this area, great progress has been made in terms of treatment methods and surgical techniques. Axis screws provide important stability for both atlantoaxial fusion and lower cervical fixation. The screws commonly used for C2 fixation include lateral mass screws (1), transarticular C1–C2 screws (2, 3), and C2 pedicle screws (4), all of which have the risk of damaging the vertebral artery and may lead to serious complications of cerebral infarction (5–7). Wright (8) first proposed the use of C2 laminar screws for posterior cervical fixation in 2004. C2 laminar screws not only provide strong biomechanical fixation but also avoid damaging vertebral arteries, and relevant clinical studies have shown good clinical results (9, 10).

However, some studies have shown that Wright's proposed fixation technique carries the risk of C2 laminar screws penetrating the anterior cortex of the vertebral lamina and protruding into the spinal canal (9, 11). Unfortunately, this situation is not easily detected during intraoperative fluoroscopy and x-ray examination. Only after surgery CT scans can identify the violation of the cortical layer within the vertebral lamina and protruding into the spinal canal. Jea (11) proposed an improved technique for C2 lamina bicortical screw placement in 2007 in which an “outlet” hole was added at the tail of the lamina to reduce the risk of screw protrusion into the spinal canal. In clinical practice, we have found that due to the inability to ensure that the direction of the C2 laminar screws is along the “outlet” hole with manual, repeated attempts may be necessary. During this process, there is still a risk of protruding into the vertebral canal, and this risk may be greater for inexperienced physicians.

To address this issue, our center has designed a screw placement assistance tool that can further improve the accuracy of C2 laminar screw placement and avoid screw damage to the vertebral artery and protrusion into the vertebral canal. This section aims to clarify the core objectives of our study, including verifying the efficacy of the application of screw insertion auxiliary tools in bilateral cross C2 laminar screws.

## Materials and methods

### The C2 laminar screw placement aid tool

**Figure 1:**
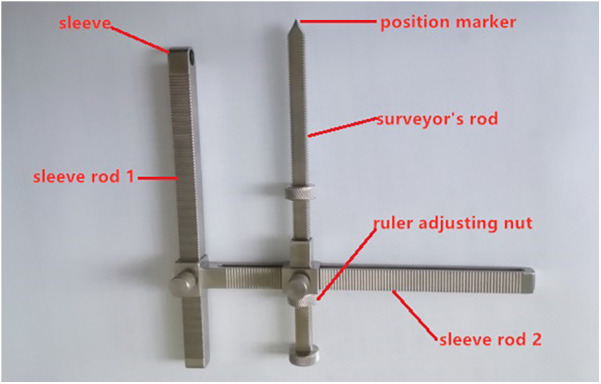
C2 laminar screw placement aid tool.

The purpose of designing screw placement aids is to help doctors determine the direction of screw placement during surgery, avoid screw protrusion into the spinal canal or damage the anterior bone cortex. This is a C2 laminar screw auxiliary tool independently designed by our center and is mainly composed of a surveyor's rod and 2 sleeve rods. The length of the surveyor's rod can be adjusted, and when in use, the surveyor's rod and sleeve rod 1 are parallel. The position marker at the far end of the surveyor's rod and the center of the sleeve of sleeve 1 should be in a straight line. The sleeve design at the far end of sleeve 1 is the “inlet” position of the screw, and the circular position marker at the far end of sleeve 2 is the “outlet” position of the screw see Figure 1 for details.

### Patients and clinical data

From March 2021 to February 2022, 6 patients with upper cervical spine injuries who required open reduction fusion internal fixation surgery were treated in our center, including 4 males and 2 females. The age range was 23–47 years, with an average age of 35 years. The cause of injury in 6 patients was traffic accidents. All patients underwent x-ray and CT examinations before surgery and were diagnosed with odontoid fracture. Some patients had atlantoaxial joint dislocation or spinal cord injury. The detailed information is shown in Table 1.

**Table 1:** Data of patients who underwent C2 bicortical laminar screw fixation.

Age	Sex	Causes of damage	Damage characteristics
28	Female	Traffic accident	Type II^a^ odontoid process fracture, forward dislocation of Atlanto-axial joint
32	Male	Traffic accident	Type II^a^ odontoid process fracture, posterior dislocation of Atlanto-axial joint
23	Male	Traffic accident	Type II^a^ odontoid process fracture, forward dislocation of Atlanto-axial joint
43	Female	Traffic accident	Type II^a^ odontoid process fracture, forward dislocation of Atlanto-axial joint
37	Male	Traffic accident	Type III^a^ odontoid process fracture, posterior dislocation of Atlanto-axial joint
47	Male	Traffic accident	Type II^a^ odontoid process fracture, cervical spinal cord hyperextension injury (Frankel D), cervical canal stenosis

a Dentiform process fractures are classified according to the Anderson classification system.

### Surgical technique

**Figure 2:**
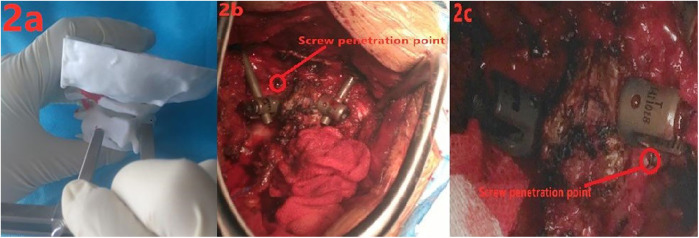
(**a)** demonstration of the use of a screw placement tool on a 3D model; **(b,c)** intraoperative placement of two bicortical screws in the axial lamina.

All patients underwent induction of general anesthesia, the head was placed on a headrest to maintain a neutral position between the head and cervical spine. The surgical approach involved a posterior median incision to expose the spinous process, vertebral lamina, lateral mass, and pedicle stenosis of C1 and C2 layer by layer. It was determined whether to expose the spine downward or the occipital bone upward as needed. Following the surgical technique introduced by Wrigt (12), a needle was inserted at the junction of the C2 vertebral lamina and spinous process. With the help of our screwplacement assistance tool, the sleeve of sleeve 1 was positioned at the ideal needle insertion position (entrance), and the length of sleeve 2 was adjusted to ensure that the position marker of the surveyor's rod was in the appropriate position (exit), as demonstrated on a 3D printed model (Figure 2A). The insertion point and direction were determined, and a hand drill was used to make an opening at the entrance to the position marker of the surveyor's rod and then slowly advanced into the vertebral plate until it penetrated the bone cortex at the exit. After measurement, a 3.5 mm screw was inserted for fixation (13), and the opposite laminar screw was inserted again according to the above operation (Figures 2B,C). After the bicortical screws were inserted crosswise on both sides of the vertebral lamina, titanium rods were used to connect and lock the occipital or atlantoaxial screws, and bone grafting was performed if necessary.

## Results

The surgical duration ranged from 1.8–2.3 h, with an average of 2 h. No infection occurred, and the wounds healed successfully. Postoperative CT scans showed that a total of 12 C2 bicortical lamina screws were implanted in 6 patients. The axial lamina screws were well positioned and located in the center of the axial lamina, without any deviation or detachment. There were also no cases in which screws penetrated the medial cortical bone of the lamina or protruded into the spinal canal. No patients experienced spinal cord nerve or vertebral artery injury (iatrogenic injury), and one patient with preoperative spinal cord injury had spinal cord function restored from Frankel D to Frankel E after surgery. The 6 patients in this study were followed up for 9–16 months, with an average of 11.5 months. The results showed satisfactory fracture reduction and good stability in all patients, and the fractures healed successfully.

### Typical case

**Figure 3:**
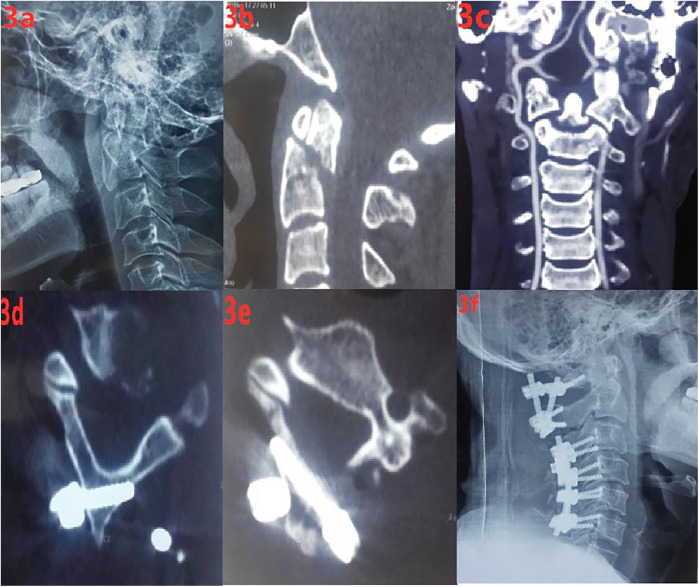
(**a)** preoperative cervical lateral x-ray; **(b,c)** preoperative cervical spine CT; **(d,e)** postoperative CT showing that the C2 laminar screws were in good position and did not protrude into the spinal canal; **(f)** postoperative lateral x-ray of the cervical spine.

A 47-year-old male patient sustained an upper cervical spine injury due to a traffic accident. After x-ray and CT examinations were completed, the diagnosis was (1) Odontoid process fracture (Anderson type II), (2) Cervical spinal cord hyperextension injury (Frankel D), and (3) Cervical canal stenosis. Posterior open reduction, bone grafting, fusion, and internal fixation with bicortical laminar screws were planned for the odontoid process fracture. Below is the detailed information of the case (Figures 3A–F).

## Discussion

In posterior fixation of the upper cervical spine, the axis provides an important stable foundation, and with continuous developments in technology, surgical methods are constantly improving. Early fixation methods include the Gallie technique, Brooks technique (14), and their improved methods, which have poor stability and high failure rates (15). A clinical study revealed that the incidence of vertebral artery injury during transarticular screw placement surgery is 4% (16). Pedicle screws also have good fixation strength and stability, but for some patients whose pedicles are too small, it may not be possible to use pedicle screws for fixation, or the risk is high (17, 18). The results of a cadaver study also confirmed this, with 20% of vertebral specimens having a pedicle diameter less than 3.5 mm (19). In addition, pedicle screws also pose a risk of damaging the vertebral artery. In 2004, Wright first proposed C2 laminar screw placement, which can avoid damaging the vertebral artery and provide strong fixation strength. However, subsequent studies have shown that this technique carries the risk of screws penetrating the inner wall of the vertebral lamina and protruding into the spinal canal (11). To solve this problem, our center has designed a screw placement assistance tool to help surgeons insert C2 vertebral lamina screws. Long-term application has revealed its good clinical effect and good clinical value.

The axis is different from that of other vertebrae in that its lamina is thicker (20), providing a prerequisite for the fixation of lamina screws. Compared to Magerl transarticular screws and pedicle screws, there is almost no risk of vertebral artery injury with lamina screws, and most of the procedure can be performed under direct vision (9). However, during the process of the screw entering the vertebral lamina, the surgeon cannot observe the depth and direction of the screw with the naked eye or on intraoperative fluoroscopy images because the relative position of the screw and the vertebral canal is difficult to determine through anteroposterior and lateral radiographic images, and there is a risk of the screw penetrating the inner wall of the vertebral lamina and protruding into the vertebral canal, causing damage to the dura mater and spinal cord (21). A study by Parker et al. (22) reported that the incidence of vertebral plate rupture caused by C2 vertebral lamina screws was 1.3%. Previous clinical studies (23) have suggested that the direction of the screw path should be parallel to the contralateral vertebral plate plane during the drilling process, but this may affect the fixation strength of the screw and be difficult to objectively quantify. Jea (11) proposed a bicortical lamina screw fixation technique, which involves opening a window at the exit point and looking directly at the screw inlet and outlet points. This approach can reduce the risk of screw damage to the spinal canal and achieve cross fixation of two screws with bicortical fixation. In addition, we believe that bicortical fixation provides greater fixation strength than monocortical fixation because the pedicle is surrounded by cortical bone. The vertebral lamina only has cortical bone in the front and back, while cancellous bone is present in the upper and lower parts. Therefore, the risk of screw displacement in the vertebral lamina is greater, and bicortical fixation may reduce this risk. Although this technology can be used to observe the entry and exit points of screws, the direction of screw travel within the vertebral lamina cannot be accurately predicted and judged, thereby requiring subjective judgment by the surgeon and potentially repeated attempts. Even if the final screw penetrates from the predetermined exit point, it may have caused damage to the inner wall of the vertebral lamina and spinal canal during the attempt. We can not only ensure that the C2 vertebral lamina screw penetrates from the predetermined exit point but we can also predict the direction of the screw will travel within the vertebral plate, thereby preventing the screw from damaging the inner wall of the vertebral plate. In our study, none of the 6 patients experienced any screw damage to the inner wall of the vertebral plate or vertebral canal.

The biomechanical results showed that the strength and pullout resistance of C2 laminar screws were comparable to those of transarticular screws and pedicle screws (12, 24). This means that for patients for whom C1/2 transarticular screw placement and pedicle screw placement are considered high-risk procedures, posterior fixation with C2 lamina screws may be a good alternative option. There were no intraoperative or postoperative complications in Wright's first report on axial lamina screw placement, and no cases of cervical instability were found on flexion or extension x-ray at 6 weeks after surgery (8). A study on the early clinical results of C2 lamina screw placement by Wang et al. showed that 11 of 30 patients had screw damage to the inner wall of the vertebral plate. Fortunately, none of these patients had neurological symptoms (9). A comparative study (22) between C2 lamina screw placement and pedicle screw placement by Parker et al. showed that the incidence of secondary surgery due to screw loosening one year after surgery was 6.1%, and the incidence of surgery involving pedicle screws was 0%. In our study, no patients experienced loosening or failure of screws or secondary surgery, and all patients' fractures were satisfactorily reduced and ultimately healed successfully.

C2 laminar screw placement does not rely on the position of the pedicle, lateral mass, or transverse foramen. As the vertebral plate is located behind the axis, the screw path of the laminar screw is far from the transverse foramen, so there is almost no risk of damaging the vertebral artery (8, 11). In addition, C2 laminar screws are placed under direct vision and do not require intraoperative fluoroscopy, thereby avoiding additional radiation effects on medical personnel. C2 laminar screw placement, as a technique for posterior fixation of the high cervical spine, can be a good choice when transarticular screw placement and pedicle screw placement are difficult.

## Conclusion

Destruction of the inner wall of the vertebral lamina or invasion of the vertebral canal by screws is the most common complication of axial vertebral lamina screw placement. Our designed screw placement assistance tool can help surgeons safely and accurately insert bicortical cross screws into the axial vertebral lamina, avoiding screw damage to the inner wall of the lamina and spinal canal. Of course, as this is a new design tool, more clinical studies are needed to confirm its effectiveness.

## Data Availability

The original contributions presented in the study are included in the article/Supplementary Material, further inquiries can be directed to the corresponding authors.
